# PAS domain containing regulator SLCG_7083 involved in morphological development and glucose utilization in *Streptomyces lincolnensis*

**DOI:** 10.1186/s12934-023-02263-3

**Published:** 2023-12-13

**Authors:** Chun-Yan Lin, Yixian Ru, Yanchao Jin, Qi Lin, Guang-Rong Zhao

**Affiliations:** 1 College of Materials and Chemical Engineering, Minjiang University, Fuzhou, 350108 China; 2 Frontier Science Center for Synthetic Biology and Key Laboratory of Systems Bioengineering (Ministry of Education), School of Chemical Engineering and Technology, Tianjin University, Yaguan Road 135, Jinnan District, Tianjin, 300350 China; 3 SynBio Research Platform, Collaborative Innovation Centre of Chemical Science and Engineering (Tianjin), Tianjin University, Yaguan Road 135, Jinnan District, Tianjin, 300350 China; 4 Georgia Tech Shenzhen Institute, Tianjin University, Dashi Road 1, Nanshan District, Shenzhen, 518055 China; 5 College of Environmental and Resource Sciences, Fujian Normal University, Fuzhou, Fujian 350117 China

**Keywords:** *Streptomyces lincolnensis*, PAS domain, Transcriptional regulator, Morphological development, Glucose utilization

## Abstract

**Background:**

*Streptomyces lincolnensis* is well known for producing the clinically important antimicrobial agent lincomycin. The synthetic and regulatory mechanisms on lincomycin biosynthesis have been deeply explored in recent years. However, the regulation involved in primary metabolism have not been fully addressed.

**Results:**

SLCG_7083 protein contains a Per-Arnt-Sim (PAS) domain at the N-terminus, whose homologous proteins are highly distributed in *Streptomyces*. The inactivation of the *SLCG_7083* gene indicated that SLCG_7083 promotes glucose utilization, slows mycelial growth and affects sporulation in *S. lincolnensis*. Comparative transcriptomic analysis further revealed that SLCG_7083 represses eight genes involved in sporulation, cell division and lipid metabolism, and activates two genes involved in carbon metabolism.

**Conclusions:**

SLCG_7083 is a PAS domain-containing regulator on morphological development and glucose utilization in *S. lincolnensis*. Our results first revealed the regulatory function of SLCG_7083, and shed new light on the transcriptional effects of SLCG_7083-like family proteins in *Streptomyces*.

**Supplementary Information:**

The online version contains supplementary material available at 10.1186/s12934-023-02263-3.

## Background

Streptomycetes are well known for their abundant secondary metabolites that are significant resource of candidate drug, such as antibacterial, antifungal, antiparasitic, anticancer, and immunosuppress agents [1]. Their complex life activities, including spore germination, mycelial growth and differentiation, primary and secondary metabolism, are strictly and precisely regulated by multiple levels of intertwined regulation [2]. The first step in the regulatory process is to sense physical or chemical signals, which perform by signal sensors.

The Per-Arnt-Sim (PAS) domain was first discovered in eukaryotes and named after three proteins: period circadian protein (Per), aryl hydrocarbon receptor nuclear transporter protein (Arnt) and single-minded protein (Sim). In various organisms, the PAS domain of proteins plays an important role in signal transmission and cellular regulation. PAS domain exists in many proteins and can bind with a variety of ligands, leading to the domain triggering specific cellular reactions or making proteins containing this domain sensitive to additional physical or chemical signals. Different PAS protein has the ability to sense redox potential, light, oxygen, energy level, carboxylic acid, fatty acid and other stimuli, and participates in cell development, virulence, sporulation, hypoxia adaptation, circadian rhythm, metabolism, and gene regulation and expression [3]. The PAS domains detect sensory input signals by binding cofactors or ligands. When stimulated, this domain can mediate or regulate protein-protein interactions by binding to cofactors in its hydrophobic center. Like other signal transduction systems, proteins containing PAS domains are modular: PAS sensor (input) domains detect various physical and chemical stimuli and correspondingly regulate the activity of effector (output) domains, such as catalysis or binding to DNA. PAS protein is usually intrasellar, but it can monitor the external and internal environment. One way for prokaryotic PAS proteins to sense the environment is to detect changes in the electron transport system as an early warning system for the decline of cell energy level. The PAS proteins usually combine with DNA binding domain, forming transcription regulators in prokaryotic. A PAS domain-containing regulator with a YheO-like PAS6 domain linked to a helix-turn-helix domain was identified in *Campylobacter jejuni*, which modulated the flagella-flagella interactions [4]. The PAS-LuxR regulator PteF cross regulates the biosynthesis of filipin and oligomycin in *Streptomyces avermitilis* [5]. Another PAS-LuxR regulator PimM, controls the polyene macrolide biosynthesis by directly binding eight promoters of pimaricin genes in *Streptomyces natalensis* [6].

The PAS domain-containing protein MmyB is a positive regulator on methylenomycin biosynthesis, and encoded by methylenomycin (*mmy*) BGC located in a giant linear plasmid (SCP1) in *Streptomyces coelicolor* A3(2) [7]. MmyB is induced and activated by the extracellular signal molecule 2-alkyl-4-hydroxymethylfuran-3-carboxylic acids synthesized by *S. coelicolor* A3(2), thus initiating the synthesis of methylenomycin [8]. Besides PAS domain, MmyB also contains a xenobiotic response element (XRE) family transmembrane DNA binding domain [9]. The MmyB homologous proteins are widely distributed in actinomycetes and belong to a new transcription factor family, which all contain PAS domains named MmyB domains. MltR (PDB ID 3pxp) (Caur_2278) is the first protein with determined crystal structure in the MmyB transcription factor family, and it was reported to bind to fatty acid (myristic acid) ligand [10]. The active form of MLTR is homodimer, in which each monomer is composed of an N-terminal DNA binding domain and a PAS related domain involved in ligand binding at the C-terminal. Sequence analysis shows that the N-terminal DNA binding domain of MLTR contains a helix turn helix (HTH) structure, also belonging to the XRE transcription factor family. The C-terminal of MLTR is composed of a profilin-like fold core containing six strands that form an antiparallel β-sheet and nine α-helix, similar to the prototypical PAS domain. Bioinformatics analysis showed that MmyB family homologous proteins were related to antibiotics and fatty acid metabolism in actinomycetes. The homologous gene *SGR_ 6891* in *Streptomyces griseus* is adjacent to A factor synthetase gene *asfA,* and is induced by A factor (γ-butyrolactone) regulating antibiotic synthesis and cell differentiation [11].

*Streptomyces lincolnensis* is well known for producing the clinically important antimicrobial agent lincomycin. The genomes of *S. lincolnensis* had been sequenced in three strains, *S. lincolnensi*s NRRL 2936 [12], *S. lincolnensi*s LC-G [13] and *S. lincolnensis* B48 [14], which facilitates the related research on genome-wide insights into metabolism and regulation. LmbU, encoded by lincomycin biosynthetic gene cluster (*lmb* cluster), was reported as a significant pleiotropic transcriptional regulator in lincomycin biosynthesis by entirely activating the *lmb* cluster and regulating nineteen non-*lmb* genes involved in multi-drug flux to self-resistance, nitrate and sugar transmembrane transport and utilization, and redox metabolisms [15]. The ATP-binding cassette family F ATPase LmrC encoded by the resistance gene in *lmb* cluster, in addition to its own resistance function, also participated in the regulation of lincomycin biosynthesis by antibiotic-driven signaling cascade transduction [16]. Six regulators encoded by non-*lmb* genes were all identified to participate in regulation of the *lmb* cluster, that are the nitrogen regulator GlnR [17], the TetR family regulator SLCG_2919 [18], the developmental regulator BldD [19], the A-factor AdpA [20], the leucine-responsive regulatory protein Lrp [21] and the developmental regulator RamR [22]. In addition, they also play other regulation roles in *S. lincolnensis*: GlnR up-regulates the transcription of nitrate-specific ABC transporter and nitrate assimilation genes [17]; SLCG_2919 negatively regulates its adjacent gene *SLCG_2920* encoding a lincomycin resistance protein [18]; BldD positively regulates the sporulation [19]; AdpA activates the *bldA* gene for the cascade regulation of lincomycin biosynthesis [20]; Lrp is transcriptionally self-inhibited and activates its adjacent gene *SLCG_3127* encoding LysE protein; RamR affects cell growth in *S. lincolnensis* [22]. Besides, the redox-sensing regulator Rex directly controls the expression of g*lnR* and *ramC*, to indirectly regulate lincomycin biosynthesis and morphology, respectively [23].

In this study, to investigate PAS domain containing proteins in *S. lincolnensis*, we conducted the bioinformatic analysis and found the SLCG_7083 protein, whose homologous proteins widely spread in *Streptomyces* and belong to a new regulator family. We constructed the *SLCG_7083* deletion strain to comprehensively understand the regulatory effects of SLCG_7083. And then we performed the comparative transcriptomic analysis andwhich further proved the regulatory effects of SLCG_7083 on morphological development and glucose utilization.

## Results and discussion

### *In silico* analysis of the putative pleiotropic regulatory function of SLCG_7083

**Fig. 1:**
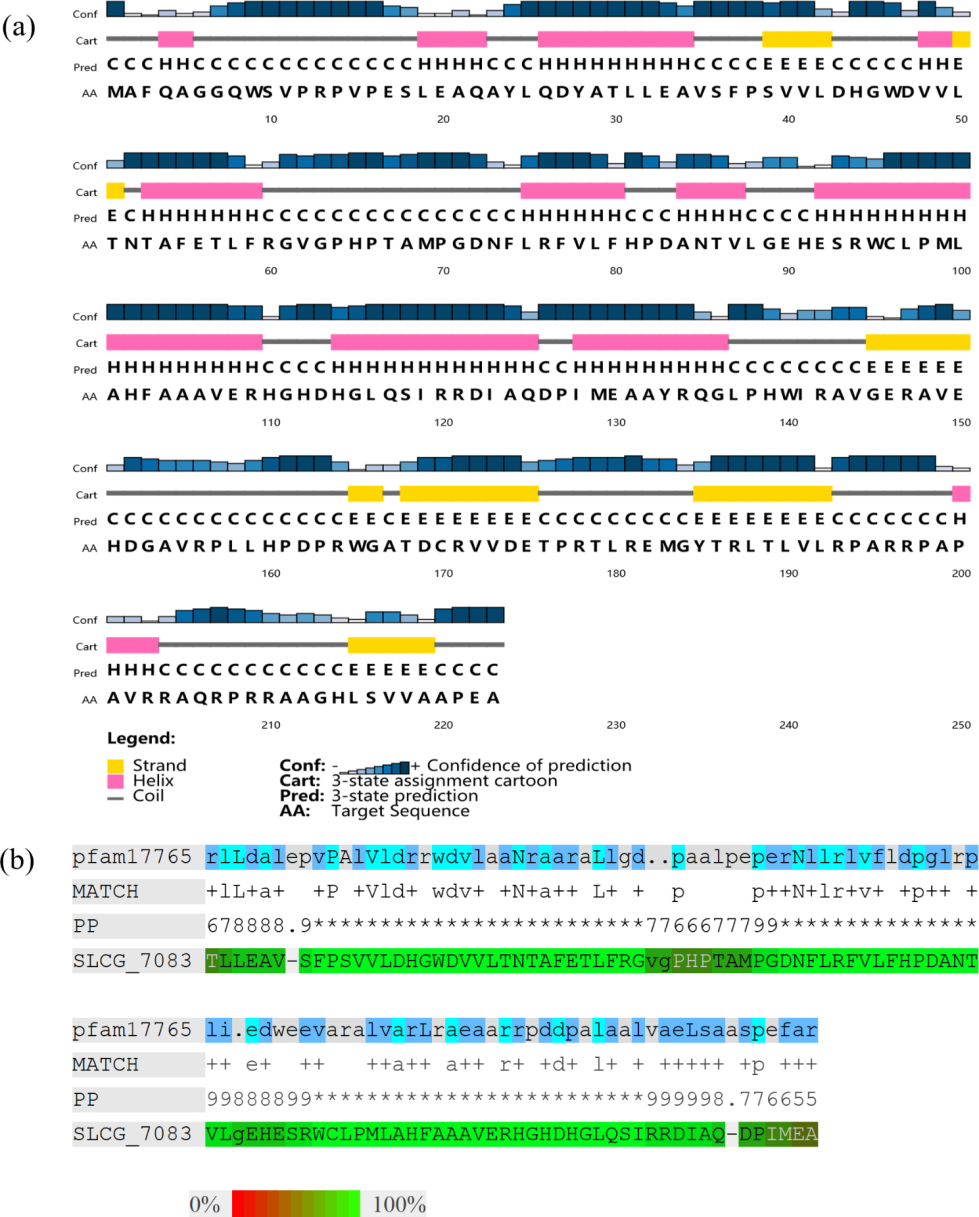
Protein domain prediction of SLCG_7083. (**a**) The secondary structure of SLCG_7083 predicted by PSIPRED software. (**b**) Amino acid sequence alignment of SLCG_7083 with the MmyB-like transcription regulator ligand binding domain (pfam17765). PP: confidence of residue per comparison. 0 represents 0–5%, 1 represents 5–15%, and so on; 9 represents 85–95%, while “*” represents 95–100% confidence

For searching the PAS domain, a protein (SLCG_7083) with 223 amino acids was found in *S. lincolnensis*. The secondary structure predicted by PSIPRED software (http://bioinf.cs.ucl.ac.uk/psipred/) showed that the SLCG_7083 protein contains multiple α helices and β folds at the C-terminator. As shown in Fig. 1a, the main structure of SLCG_7083 is distantly homologous to the conserved PAS domain for ligand binding in the MmyB-like family transcription regulator (pfam17765), which contains 168 amino acids. In addition, there was 72.55% similar between SLCG_7083 (30 to 131 amino acid residues) and pfam17765 (7 to 107 amino acid residues) (Fig. 1b). Similar to PAS domain, four β folds and a α helices/β folds were predicted in 132 to 223 amino acid residues of SLCG_7083 protein. Protein secondary structure prediction showed that the SLCG_7083 protein contains PAS domain similar to MmyB-like family transcription factors, which may be involved in identification of signal molecules.

SLCG_7083-like proteins with similarity over 45% were collected by BLASTP search against NCBI database using SLCG_7083 protein as query sequence. Then 56 SLCG_7083-like proteins were used to construct a phylogenetic tree (Fig. S1). The phylogenetic tree showed that the SLCG_7083-like proteins are highly distributed regulators in *Streptomyces* (Fig. S2), and are conservative with a PAS domain (smart00091). Most of the proteins were located on the branches containing SLCG_7083 in *S. lincolnensis*, which were separated from the branch of DicA considered to be a transcriptional inhibitor. Altogether these results indicated that the SLCG_7083-like proteins may belong to a new regulator family with PAS domain to sense signal molecules.

### Functionality of SLCG_7083 regulating mycelial growth and sporulation

**Fig. 2:**
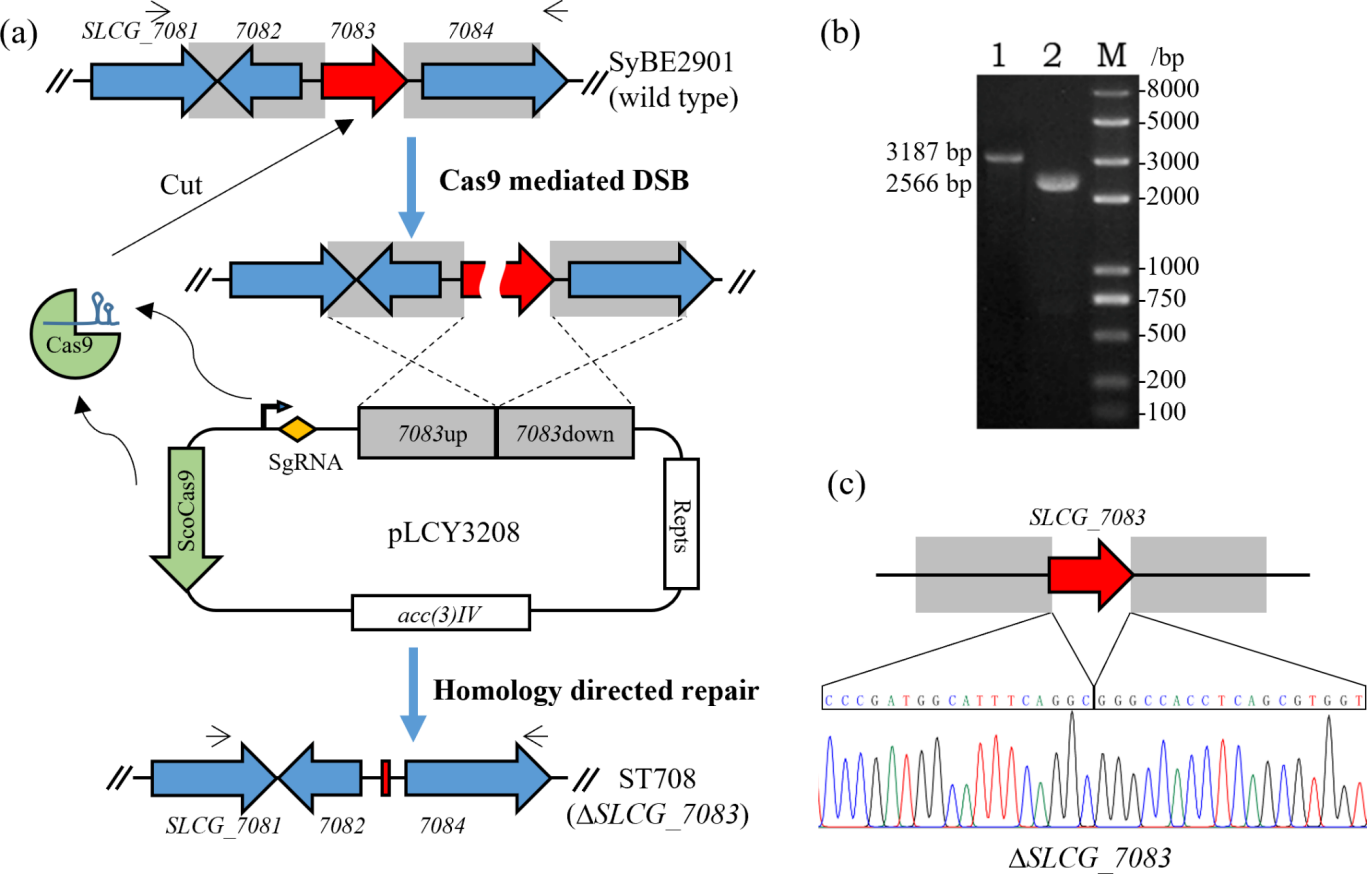
Construction of *SLCG_7083* disruption mutant ST708. (**a**) Schematic representation of the CRISPR/Cas9-mediated cleavage of genomic DNA and homology directed repair (HDR) to delete of *SLCG_7083*. (**b**) confirmation of the *SLCG_7083* deletion strain ST708 by PCR amplification. Lane 1: a 3187-bp amplicon using the original SyBE2901 genomic DNA as a template; Lane 2: a 2566-bp amplicon using the ST708 genomic DNA as a template; M: DNA ladder. (**c**) Sequencing confirmation of the amplicon from ST708 strain

**Fig. 3:**
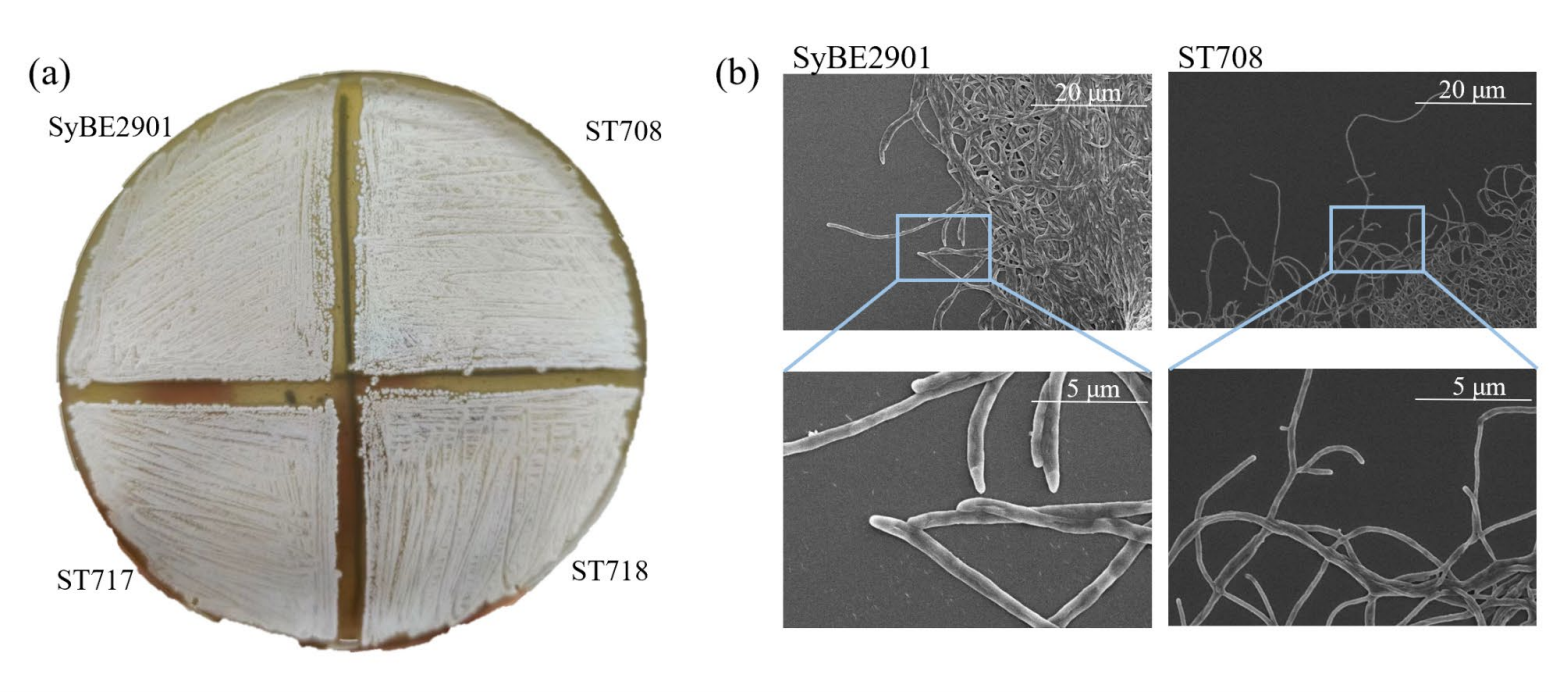
Effect of SLCG_7083 on morphological development. (**a**) Morphological observation of the original strain SyBE2901, the ΔSLCG_7083 strain ST708, the SLCG_7083 complementation strain ST718 and SLCG_7083 overexpression strain ST717 on SX medium. (**b**) Scanning electron microscope assay showing phenotypes of ST708 and SyBE2901 cultured in SM medium at 48 h, under the 2.00 k and 8.00 k magnification, respectively

To characterize the *SLCG_7083* gene, the CRISPR/Cas9-mediated in-frame *SLCG_7083* deletion strain ST708 was constructed (Fig. 2a), which was confirmed by PCR (Fig. 2b), and further verified by DNA sequencing (Fig. 2c). Moreover, the *SLCG_7083* complementation strain ST718 and *SLCG_7083* overexpression strain ST717 were also constructed. As shown in Fig. 3a, the mycelial growth and spore differentiation of the *SLCG_7083* deletion strain ST708 were slightly faster than that of the original strain SyBE2901, and the mycelial and spore quantity of ST708 was also more abundant than that of SyBE2901. And when complementing the *SLCG_7083* gene, that all restored. Overexpressing of the *SLCG_7083* gene showed no obvious difference to SyBE2901. when cultured in liquid medium, the mycelia of ST708 strain were fluffier and slower settling, compare to that of SyBE2901 strain. The scanning electron microscope assay further indicated that the mycelia of ST708 strain were thinner than that of SyBE2901 (Fig. 3b). These results indicated that knocking-out the *SLCG_7083* gene promoted the mycelial morphology and sporulation, and formed thinner mycelia in *S. lincolnensis*.

### Functionality of *SLCG_7083* regulating glucose utilization

**Fig. 4:**
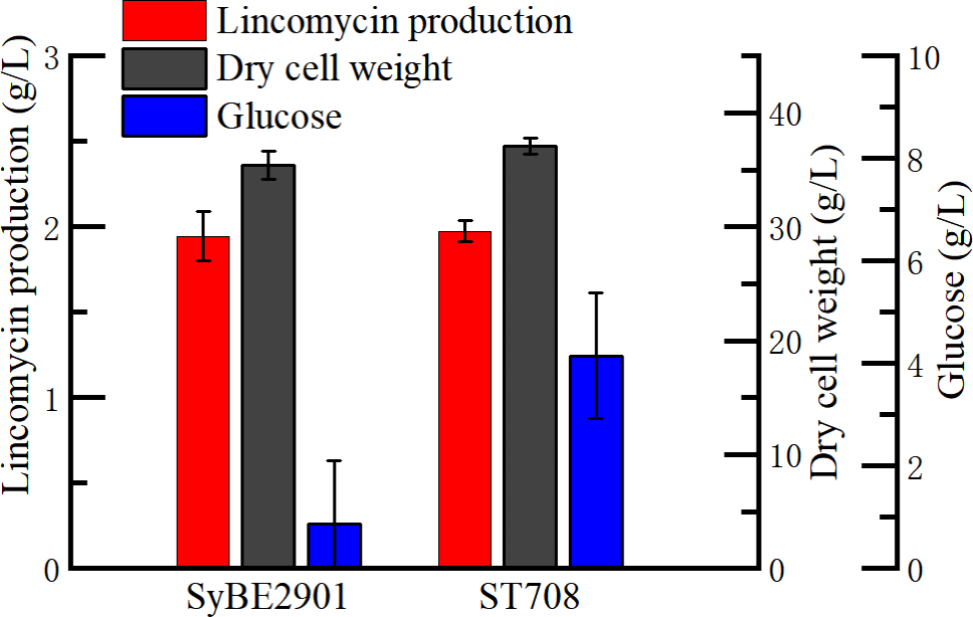
Residual glucose, dry weight and lincomycin production in fermentation broth of strains SyBE2901and ST708.

The fermentations of *SLCG_7083* deletion strain ST708 and the original strain SyBE2901were conducted and the lincomycin productions in broth were measured by HPLC. As shown in Fig. 4, with 100 g/l initial glucose, only 0.87 g/l glucose was left in the fermentation broth of SyBE2901, while 4.15 g/l glucose was left in the fermentation broth of ST708, which was higher than the residual glucose of SyBE2901, indicating that deleting *SLCG_7083* gene reduced the consumption of glucose. The dry weight of SyBE2901 and ST708 were 35.43 g/l and 37.09 g/l, respectively, showing the biomass was increased by 4.68% after deleting *SLCG_7083* gene, which indicated that the deletion of *SLCG_7083* gene was beneficial to the growth of *S. lincolnensis*. The yield of ST708 was almost equality to that of SyBE2901, indicating that this gene knockout had no significant effect on the lincomycin production. The results indicated that besides mycelial growth, SLCG_7083 may also affect glucose utilization in *S. lincolnensis*.

In order to further elucidate the function of SLCG_7083 on glucose utilization, fermentation was conducted with different concentration of initial glucose. The *SLCG_7083* deletion strain ST708 and original strain SyBE2901 were cultured in fermentation medium containing 4%, 6%, 8%, 10% and 12% glucose, respectively. Fermentation broth samples were collected at different times to detect the yield, dry cell weight and residual glucose.

**Fig. 5:**
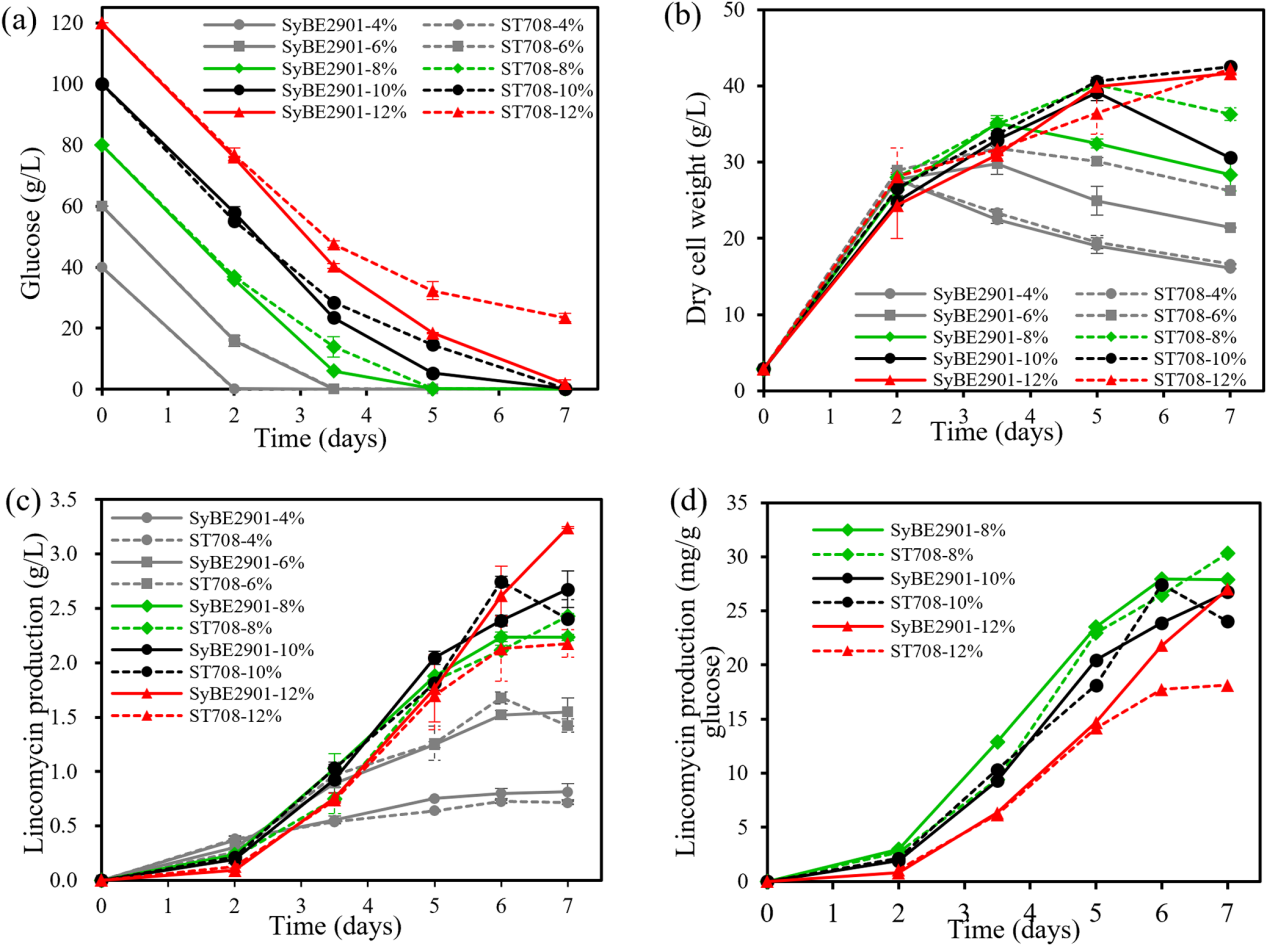
Residual glucose (**a**) in fermentation, dry cell weight (**b**), lincomycin production (**c** and **d**) and of strains SyBE2901and ST708 with different initial concentrations glucose

The curves of glucose concentration during fermentation with 4%~12% initial glucose concentration were shown in Fig. 5(a). Under the condition of low initial glucose concentration (4% and 6%), the glucose consumption of ST708 and was basically same to that of SyBE2901 (the curve coincided), and they were all consumed in the early stage of fermentation (2th or 3.5th day); Under the condition of medium initial glucose concentration (8% and 10%), the glucose consumption rate of ST708 was slower than that of SyBE2901, and was consumed on the 5th and 7th day respectively; Under the condition of high initial glucose concentration (12%), 23.52 g/L glucose remained in strain ST708 on the 7th day, while only 1.91 g/L glucose remained in strain SyBE2901. This result indicated that *SLCG_7083* gene deletion slowed down the consumption of glucose, which was consistent with the above results.

The growth curves of ST708 and SyBE2901 were shown in Fig. 5(b), and analyzed in combination with the glucose concentration curve in Fig. 5(a). When glucose was sufficient, the biomass of ST708 and SyBE2901 increased with time, but when glucose was insufficient, the biomass decreased. Under the initial glucose concentration of 6%, 8% and 10%, the *SLCG_7083* deletion strain ST708 had relatively sufficient carbon source and higher biomass than the original strain SyBE2901.

As shown in Fig. 5(c), the lincomycin yield of SyBE2901 increased as the increasing of initial glucose concentration. When the initial glucose concentration was 4%, 6% and 8%, the lincomycin yield of *SLCG_7083* deletion strain ST708 all reached the highest on 6th day. When the initial glucose concentration was increased to 10%, the lincomycin yield of ST708 reached the highest point on 6th day, which was even higher than that of SyBE2901 on 7th day. When the initial glucose concentration was increased to 12%, the lincomycin yield of SyBE2901 was still increasing due to the availability of sufficient carbon source, and the maximum yield reached 3.24 g/L on day 7, which was 21% higher than that of 10% initial glucose. Due to the abundant carbon source, the biomass of ST708 was increasing, and the mycelia was more inclined to grow by themselves and synthesize primary metabolites, resulting in relatively slowing down the synthesis of lincomycin, which is a secondary metabolite of *S. lincolnensis*.

Glucose is the main component of the fermentation medium, which provides carbon source and energy for *S. lincolnensis*. Since the initial glucose concentration was different, the yield of lincomycin was reanalyzed in combination with glucose consumption, and the amounts of lincomycin synthesis per unit glucose consumption were calculated shown in Fig. 5(d). Since the yield of lincomycin was not high with low initial concentration of glucose, only the initial glucose concentration of 8%, 10% and 12% was analyzed. According to the Fig. 5(d), on day 7, the amounts of lincomycin synthesized by SyBE2901 per unit glucose consumption all reached the highest with the initial glucose concentration of 8%, 10% and 12%, and the yields were basically equivalent. With 8% initial glucose concentration, SLCG_7083 deletion strain ST708 could synthesize 30.35 mg lincomycin per gram of glucose consumption on day 7, which was the highest yield of lincomycin compared to that with 10% and 12% initial glucose concentration. This result indicated that, for lincomycin production, deletion of SLCG_7083 gene could achieve more higher utilization rate of glucose.

In conclusion, the fermentation experiments with different initial glucose concentrations showed that the *SLCG_7083* gene was more likely to affect glucose utilization and mycelial growth.

### Comparison of transcriptomes between the original strain and the *SLCG_7083* deletion strain

To obtain insight into regulatory mechanism of SLCG_7083 protein on glucose utilization and mycelial growth and differentiation, comparative transcriptional analysis was conducted between the *SLCG_7083* deletion strain ST708 and the original strain SyBE2901. To obtain insight into the changes in gene expression levels between SyBE2901 and ST708, RNA was isolated and subjected to whole-transcriptome sequencing via ssRNA-seq. Approximately 12.82 ~ 14.73 million 150 bp paired-end clean reads (1.92 ~ 2.21G data) per sample were obtained after cleaning and checking the reads quality. Approximately 95.21%~98.91% of clean reads were aligned uniquely to the *S. lincolnensis* genome. The correlation clustering among the two biological replicates of each sample was conducted based on the expression level of all genes. All biological replicates (three RNA samples for each strain) showed correlation coefficients over 0.8, indicating good reproducibility between biological replicates. To investigate transcriptional regulation effects of SLCG_7083, the whole transcriptomes were compared.

**Fig. 6:**
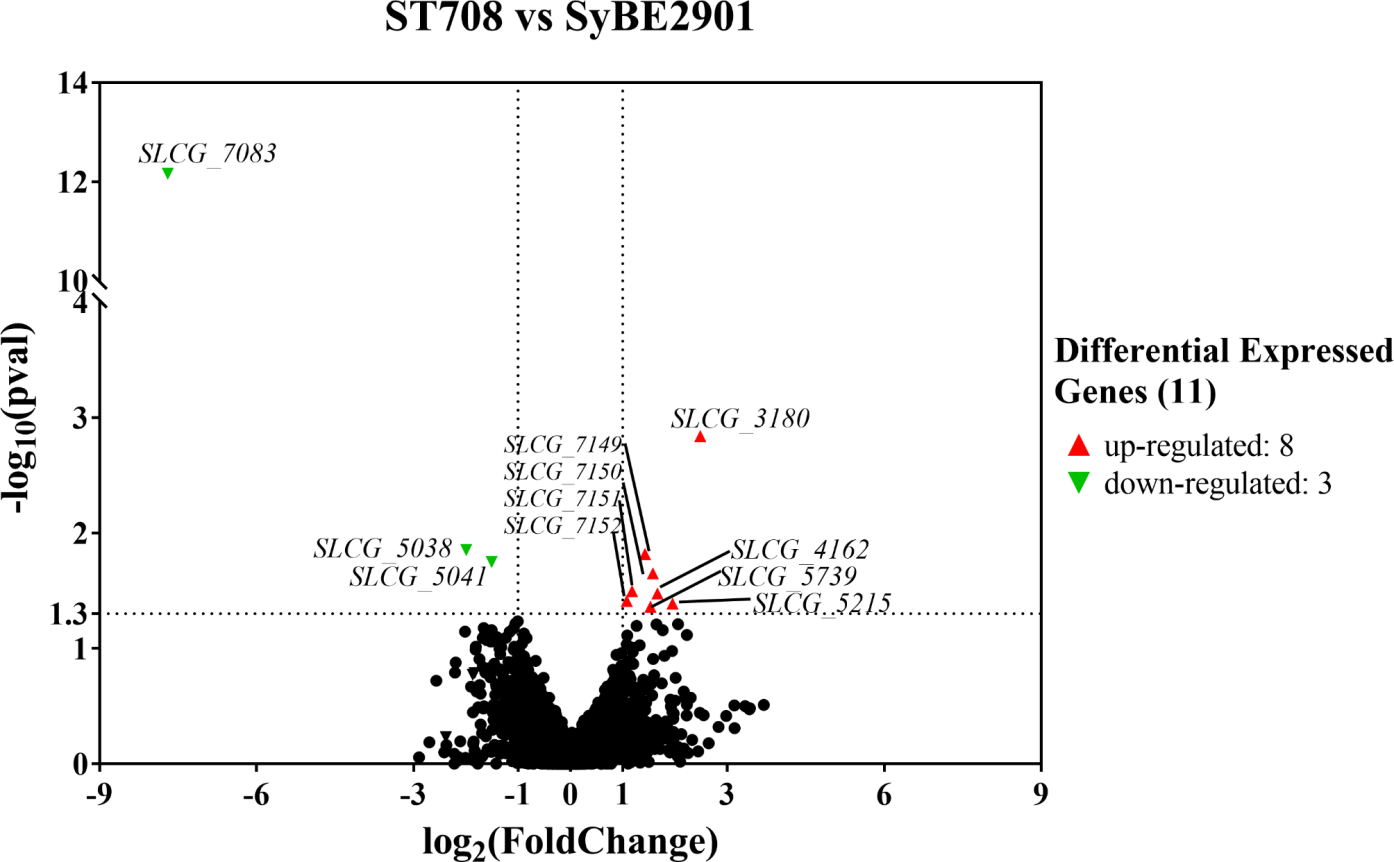
Volcano blots to show significant changes in gene expression between strain SyBE2901 and ST708.

Volcano plot was presented to identify genes with both high fold change and significance between the original strain SyBE2901 and the *SLCG_7083* deletion strain ST708 (Fig. 6). Compared to SyBE2901, the transcription expression of 11 genes were statistically different in the ST708. No reads mapped to the *SLCG_7083* gene showing the successful deletion of the *SLCG_7083* in strain ST708. Besides *SLCG_7083* gene, 2 genes displayed decreased transcription levels, and 8 gene displayed increased transcription level in the ST708 compared toSyBE2901. Of which, six genes were selected to perform semi-quantitative PCR analysis (Fig. S3) of the original strain SyBE2901, the SLCG_7083 deletion strain ST708, and the SLCG_7083 complemention strain ST718. semi-quantitative PCR analysis results in SyBE2901and ST708 were consistent with those of ssRNA-Seq. Furthermore, after complete SLCG_7083 gene to ST708, the expressions of those six were restored. Deleting SLCG_7083 did not cause transcriptional differences in *lmb* gene cluster, suggesting that SLCG_7083 didn’t control lincomycin biosynthesis, which was consistent with the previous fermentation results.

### SLCG_7083 negatively regulates two sporulation/cell division related genes

In *SLCG_7083* deletion strain ST708, the transcription of two genes (*SLCG_3180* and *SLCG_4162*) involved in sporulation and cell division increased significantly, which were 5.60 and 3.17 times higher than that in original strain SyBE2901, respectively (Table 1). This result indicated that SLCG_7083 protein affected morphological development by negatively regulating *SLCG_3180* and *SLCG_4162*.

**Table 1:** List of differentially regulated genes in the *SLCG_7083* deletion strain ST708 compared to original strain SyBE2901

Gene_id	readcount		log_2_Fold Change	pval	Description	ID/SM (%)	Origin (Protein Accession number)	EC number
	SyBE2901	ST708						
**sporulation/cell division**								
SLCG_3180	86.19	482.35	2.48	0.001449	serine/threonine-protein phosphatase, Stage II sporulation protein E	92/94	*Streptomyces mirabilis*, WP_051959054.1	3.1.3.16
SLCG_4162	44.90	142.23	1.66	0.033498	SsgA, sporulation/cell division regulator	94/97	Streptomyces viridochromogenes, WP_003999767.1	/
**Carbohydrate Metabolism**								
SLCG_5038	193.14	48.67	-1.99	0.014015	Phosphohexomutase/phosphatase	84/86	*Streptomyces cyaneochromogenes*, WP_126392030.1	3.1.3.-
SLCG_5041	335.16	118.03	-1.51	0.017798	cyclitol dehydrogenase	84/90	*Streptomyces griseofuscus*, WP_125212155.1	1.1.1.-
**Lipid transport and metabolism**								
SLCG_5215	213.11	825.51	1.95	0.040887	short-chain acyl-CoA dehydrogenase	96/98	*Streptomyces hokutonensis*, WP_019065764.1	1.3.99.12
SLCG_5739	241.29	698.13	1.53	0.043582	acetyl-CoA acetyltransferase	98/99	*Streptomyces canus*, WP_062042393.1	2.3.1.9
SLCG_7149	203.80	547.39	1.43	0.015251	acyl-CoA transferases	88/94	*Streptomyces cyaneus*, WP_128437098.1	2.8.3.-
SLCG_7150	91.39	272.56	1.58	0.022388	long-chain specific acyl-CoA dehydrogenase	94/97	*Streptomyces fulvoviolaceus*, WP_030600429.1	1.3.99.3
SLCG_7151	360.65	816.15	1.18	0.031986	acetyl-CoA acetyltransferase	98/99	*Streptomyces curacoi*, WP_062147083.1	2.3.1.9
SLCG_7152	467.97	989.21	1.08	0.038871	fatty acid oxidative multifunctional enzyme	92/96	*Streptomyces fulvoviolaceus*, WP_030600433.1	1.1.1.35 4.2.1.17 5.1.2.3

*SLCG_3180* gene encoded a serine/threonine protein phosphatase, involved in the phosphorylation on the serine/threonine and tyrosine residues of proteins in *Streptomyces* in response to developmental phases. It was predicted to be a membrane-bound protein for at least three transmembrane helical regions in its N-terminal, using the transmembrane helix prediction software TMHMM. As shown in the Fig. S4, the conserved domain analysis of SLCG_3180 showed that the 146–320 amino acid region was predicted to be stage II sporulation protein E (SpoIIE) domain (pfam07228), indicating that SLCG_3180 belonged to the characteristic SpoIIE domain phosphatase protein family. Previous report showed that SpoIIE is a bifunctional protein, which takes Mn^2+^ as a metal cofactor, and not only activates the forespore compartment-specific transcription factor σ^F^ by dephosphorylation of SpoIIAA-P, but also participates in oligomerization stabilizes FtsZ during asymmetric septum formation [24]. Therefore, it was speculated that SLCG_7083 affected the formation of correct membrane during *S. lincolnensis* sporulation and cell division by negatively regulating SLCG_3180.

*SLCG_4162* gene encoded a SsgA regulator, which belong to actinomycete specific regulatory protein family that controls cell division and spore maturation in *Streptomyces* [25]. The morpho protein SsgA controls all processes of cell wall remodeling, such as spore germination, mycelial tip growth, branching and septum formation [26]. SsgA mediated localization of SsgB to recruit the tubulin FtsZ at the position where the septum will be formed, thereby activating the cell division of sporogenesis [27]. It was reported that overexpression of *ssgA* in *Streptomyces coelicolor* and *Streptomyces lividans* led to a sharp increase in vegetative mycelium and fragmentation of the mycelial clumps, then significantly increased the growth rate [28]. This is consistent with the fermentation result of deleting *SLCG_7083* in *S. lincolnensis*.

Therefore, SLCG_7083 regulated spore formation and mycelial division by inhibiting the expression of SpoIIE and SsgA protein in *S. lincolnensis*.

### SLCG_7083 negatively regulates six genes involved in lipid metabolism

Other six up-regulated genes in *SLCG_7083* deletion strain ST708 are all involved in lipid metabolism, with 2.11 to 3.87 times transcriptions compared to original strain SyBE2901 (Table 1). They encode proteins involved in fatty acids β oxidative catabolic pathway, including a short-chain acyl-CoA dehydrogenase (SLCG_5215), two acetyl-CoA acetyltransferases (SLCG_5739 and SLCG_7151), an acyl-CoA transferases (SLCG_7149), a long-chain specific acyl-CoA dehydrogenase (SLCG_7150), a fatty acid oxidative multifunctional enzyme (SLCG_7152). In particular, four of them (*SLCG_7149* ~ *7152*) were continuously arranged in the genome.

After deleting *SLCG_7083* gene, the expression of the above six lipid metabolism genes increased, which promoted the lipid utilization, resulting in the increase of the biomass. Therefore, SLCG_7083 regulates the growth of *S. lincolnensis* by negatively regulating the lipid metabolism.

### SLCG_7083 positively regulates two carbon metabolism related genes

In contrast, after *SLCG_7083* gene being deleted, the transcription level of *SLCG_5038* and *SLCG_5041* respectively decreased to 25.20% and 35.22% of that in the original strain, demonstrating SLCG_7083 positively regulates transcription of *SLCG_5038* gene and *SLCG_5041* gene.

Bioinformatics analysis showed that SLCG_5038 protein predicted to be phosphohexomutase/phosphatase with haloacid dehalogenase domain. In *Lactococcus lactis*, the SLCG_5038 homologous protein shows mutase activity, catalyzing the mutual transfer of glucose 1-phosphate and glucose 6-phosphate. Therefore, SLCG_5038 may participate in the intracellular glycolysis pathway, tricarboxylic acid cycle, pentose phosphate pathway and other basic glucose metabolism through hexose phosphate translocation and hydrolysis of phosphate sugar.

In *SLCG_7083* deletion strain ST708, another down-regulated gene *SLCG_5041* encodes protein similar to SalM [29] from the salbostatin biosynthetic gene cluster (BGC) and AcbL [30] from acarbose BGC, with 68% and 57% similarity, respectively, which were reported to be cyclitol dehydrogenases. However, AcbL was recently shown to be cyclitol dehydratase instead [31], indicating that SLCG_5041 may also catalyze cyclitol dehydration involved in glucose metabolism.

Interesting, the *SLCG_5038* gene and *SLCG_5041* gene are all located in the predicted C_7_-cyclitol BGC, which involved in C_7_-cyclitol derivatives synthesis from sedoheptulose 7-phosphate that comes from pentose phosphate pathway. It was reported that four C_7_-cyclitol derived carbasugars were isolated from *S. lincolnensis* DSM 40,355 [32], suggesting that SLCG_5038 and SLCG_5041 were involved in C_7_-cyclitol biosynthesis in *S. lincolnensis*. Deleting *SLCG_7083*, resulted in down-regulated expression of *SLCG_5038* and *SLCG_5041* in C_7_-cyclitol BGC, which reduced use of sedoheptulose 7-phosphate as substrate, following by slowing down the consumption of glucose. Therefore, the SLCG_7083 regulator positively regulated *SLCG_5038* and *SLCG_5041* gene to affect glucose utilization, which is consistent with fermentation results above.

## Conclusions

The results of this study demonstrated that the PAS domain containing protein SLCG_7083 is a regulator not only on mycelial growth and morphological development, but also on glucose utilization in *S. lincolnensis*, by negatively regulating two sporulation/cell division related genes and six lipid metabolism related genes, and positively regulating two carbon metabolism related genes. However, the detailed regulation mechanism of SLCG_7083 were not be addressed in this study, and await further research. Our results provided evidence to elucidate the regulatory functions of SLCG_7083-like PAS domain-containing proteins in *Streptomyces*.

## Methods

### Secondary structure and phylogenetic analyses of SLCG_7083-like proteins

PSIPRED software was used to predicted the secondary structure of the SLCG_7083 protein. The SLCG_7083-like proteins were collected by BLASTp search against NCBI database, and then used to construct phylogenetic tree. Alignments and phylogenetic analysis were performed using MEGA7 [33] by the neighbor-joining method (Kimura 2-parameter model + G) [34] and 500 bootstrap replications. The GenBank accession numbers of SLCG_7083-like proteins were listed in Supplementary Table S1.

### Strains, plasmids and culture conditions

The bacterial strains and plasmids used in this study are described in Table 2. Primers used in this study are listed in Table S2. The sporulation and fermentation of *S. lincolnensis* strains were carried out as previously described [35]. Briefly, the spores of *S. lincolnensis* were routinely cultivated on the modified Gauze’s Medium No.1 for 7 days at 30 °C. For fermentation, the spores inoculated into 25 ml seed medium (2% soluble starch, 1% glucose, 3% corn steep liquor, 1% soybean, 0.15% (NH_4_)_2_SO_4_, 0.4% CaCO_3_, pH 7.1) and grown for 2 days at 30 °C, 250 rpm. Then 2 ml seed culture was added into 25 ml fermentation medium (10% glucose, 0.15% cream corn, 2% soybean, 0.8% NaNO_3_, 0.5% NaCl, 0.6% (NH_4_)_2_SO_4_, 0.03% K_2_HPO_4_, 0.8% CaCO_3_, pH 7.1) and cultivated for 7 days at 30 °C, 250 rpm. Appropriate antibiotics were added in the medium when necessary.

**Table 2:** Strains and plasmids used in this study

Strain or plamid	Characteristics	Reference or source
Strains		
*E. coli*		
DH5α	General cloning host	Invitrogen
ET12567/pUZ8002	Donor strain for intergeneric conjugation	[39]
*S. lincolnensis*		
SyBE2901	Original strain for high lincomycin-producer, derived from ATCC25466	ATCC25466
ST708	SyE2901 ∆*SLCG_7083*	This study
ST717	SyBE2901 with pLCY010-7083	This study
ST718	ST708 with pLCY010-7083	This study
Plasmids		
pKCcas9dO	*acc(3)IV*, pSG5, *tipA*-*Scocas9*, j23119, *actII-orf4* guide-RNA, homologous region flanking *act-orf4*	[36]
pLCY3208	*acc(3)IV*, pSG5, *tipA*-*Scocas9*, j23119, *SLCG_7083* guide-RNA, homologous region flanking *SLCG_7083*	This study
pLCY010	pUWL201 derivative, *amp*^*r*^, *tsr*^*r*^, *hyg*^*r*^, carrying P_ermE*_ promoter	[35]
pLCY010-7083	pLCY010 with P_ermE*_SLCG_7083	This study

### Inactivation and complementation of *SLCG_7083* gene

CRISPR/Cas9-mediated genome edition [36] was used for the *SLCG_7083* in-frame deletion. As shown in Fig. S1, the CRISPR/Cas9 editing plasmid pLCY3208 was first constructed. The Guide sequence of sgRNA targeting to *SLCG_7083* gene was 21-nt sequence “Tgccgatgctggcgcacttcg” before the protospacer adjacent motif (PAM) “CGG”, which was added to the 5’-end of primer 7083gRNA-F. Using plasmid pKCcas9do as template, the 112 bp sgRNA fragment was amplified by primers 7083gRNA-F and gTEMdn (Table S2). At the same time, the upstream homologous arm (7083-up, 1137 bp) and the downstream homologous arm (7083-down, 1107 bp) of *SLCG_7083* gene were amplified from the genomic DNA of *S. lincolnensis*, respectively. The fragments of sgRNA, 7083-up and 7083-down were fused to into *SLCG-7083* deletion cassette fragment by overlapping extension PCR, using 7083gRNA-F and 7083down-R as primers. And then the *SLCG-7083* deletion cassette fragment was digested by *Bcu* I and *Hind* III, and ligated to the same enzymes linearized pKCcas9do to yield plasmid pLCY3208.The CRISPR/Cas9 editing plasmid pLCY3208 was introduced into the original strain SyBE2901 mediated by ET12567/pUZ8002, during that genome edition was performed resulting in *SLCG_7083* gene deletion. The intergeneric conjugation was performed following the procedure described previously [37]. The mutants were identified with apramycin resistance and confirmed by PCR using primers Q7083Y-F and Q7083Y-R, and then cultivated at 37℃ for two rounds without antibiotics to cure the editing plasmid.

For complementation of *SLCG_7083* gene, the 781-bp fragment containing the *SLCG_7083* gene was amplified using 7083-F and 7083-R as primers, and ligated to pLCY010, resulting plcy010-7083. The vectors pLCY0010-7083 was introduced into the *SLCG_7083* deletion strain ST708 and the original strain SyBE2901, generating the complementation strain ST718 and overexpression strain ST717, respectively.

### Scanning electron microscope assay

Mycelia of the Δ*SLCG_7083* strain ST708 and the original strain SyBE2901 grown in SM medium (1% glucose, 0.4% yeast extract, 0.4% peptone, 0.4% K_2_HPO_4_, 0.2% KH_2_PO_4_, 0.05% MgSO_4_, pH 7.0) [37] at 30 ℃ for 48 h. Equivalent mycelia were collected and placed in 2.5% glutaraldehyde at 4 ℃ overnight. Washed twice with water and then gradient dehydration using ethanol solutions (30, 50, 70, 90, 95, and 100%). Dried naturally and sprayed with platinum. Then observed with Hitachi SU8000 scanning electron microscopy (Hitachi, Japan).

### Analysis of biomass, residual glucose and lincomycin production in fermentation broth

The dry weight of mycelia was used to measure the biomass of *S. lincolnensis*. After cultivation, the precipitate was collected from fermentation broth with centrifuge for 12,000 r/min 5 min, washed twice with water, dried in an 80 ℃ oven, and then weighed by electronic analytical balance. Meanwhile, supernatant collected from fermentation broth was used to detect the residual glucose by biosensor (SBA-90B, Shandong Academy of Sciences, China).

The analysis of lincomycin was performed as previously described [15]. Briefly, 0.4 mL supernatant was collected from fermentation broth, then mixed with 0.6 mL methanol and centrifuged again to remove the residue. The samples were filtered through 0.22 μm of nylon membrane and then subjected to HPLC analysis (Agilent 1200, USA) on C18 column (4.6 × 250 mm, 5 μm, Agela Technology, China) at UV 214 nm, 25 °C. The mobile phase was 50% ammonium acetate solution (0.005 mol l^− 1^, adjust to pH 9.0 with ammonia) and 50% methanol.

### RNA-Seq transcriptomic analysis

Strand specific RNA sequencing (ssRNA-Seq) was used to investigate transcription of *S. lincolnensis* as the procedures described previously [15]. The mRNAs were enriched from 3-day total RNAs, by Ribo-zero kit. The strand-specific RNA sequencing libraries were generated using NEBNext Ultra Directional RNA Library Prep Kit for Illumia (NEB), and then deeply sequenced on an Illumina Hiseq 2500 platform performed. The transcriptome raw data of SyBE2901 and ST708 were deposited in the NCBI Sequence Read Archive (SPA) under accession number PRJNA967154.The clean reads were aligned with the genome of *S. lincolnensis* LC-G (GeneBank ID: 1,435,096,411) by Bowtie2-2.2.3 [38]. Genes with ∣log_2_(FoldChange)∣>1 and q value < 0.05 found by DEGSeq were assigned as differentially expressed.

### Semi-quantitative PCR analysis

Primers used in semi-quantitative PCR experiments were listed in Table S2. Total RNA was isolated from 3-days *S. lincolnensis* mycelia in fermentation medium, using RNAprep pure Cell/Bacteria Kit (TianGen, China) according to the manufacturer’s protocol. Reverse transcription was conducted using HiScript II 1st Strand cDNA Synthesis Kit (+ gDNA wiper) (Vazyme, China), with the conditions set as following: 55℃ for 30 min, 85℃ for 2 min. Using gene specific primers and 500 ng of total RNA as template, the first strand was generated. For analyzing transcription, the 1st cDNA reaction mixture was diluted five times, and then used as the template to amplify ds-cDNA in the following semi-quantitative PCR. All experiments were conducted in triplicate in each case. The semi-quantitative PCR products were detected by agarose gel electrophoresis, and then exposed under UV to analyze relative intensities using densitometric analysis software.

### Electronic supplementary material

Below is the link to the electronic supplementary material.

Supplementary Material 1

## Data Availability

The datasets used and/or analyzed during the current study are available from the corresponding author on reasonable request.

## Abbreviations

PAS: Per-Arnt-Sim
BGC: Biosynthetic gene cluster
XRE: Xenobiotic response element
HTH: Helix turn helix
*lmb *cluster: Lincomycin biosynthetic gene cluster
ssRNA-Seq: Strand specific RNA sequencing
PAM: Protospacer adjacent motif
