# Supplementary material for: PAS domain containing regulator SLCG_7083 involved in morphological development and glucose utilization in Streptomyces lincolnensis
Source: Microb Cell Fact. 2023 Dec 13;22:257. doi: 10.1186/s12934-023-02263-3 (PMC10717218; doi:10.1186/s12934-023-02263-3)
Supplement: Supplementary file 1 — Supplementary Material 1 [file 12934_2023_2263_MOESM1_ESM.docx]

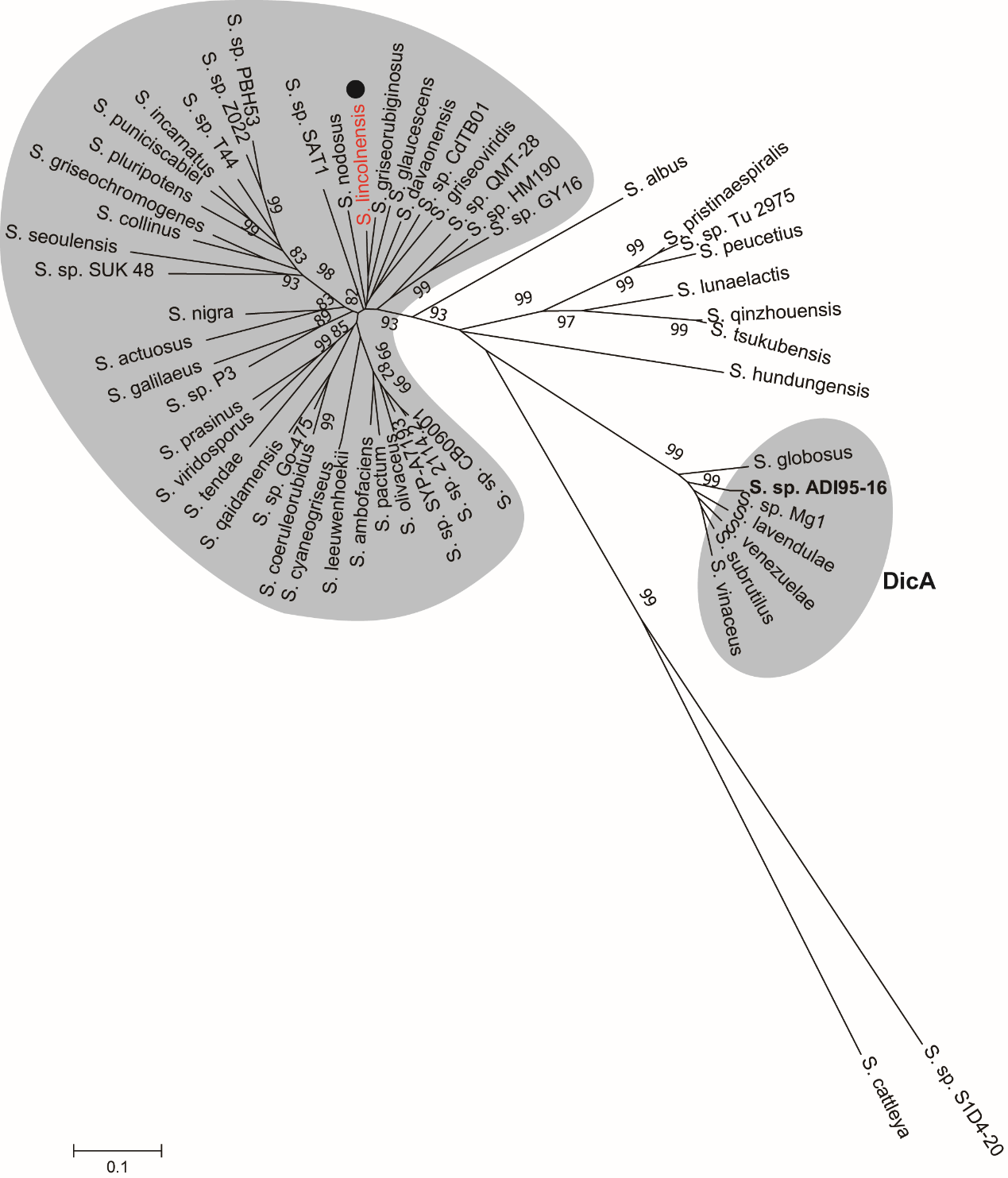


**Fig. S1** Phylogenetic analyses of SLCG_7083-like proteins from streptomycetes. The GenBank accession numbers of 56 SLCG_7083-like proteins are listed in Supplementary Table S1.


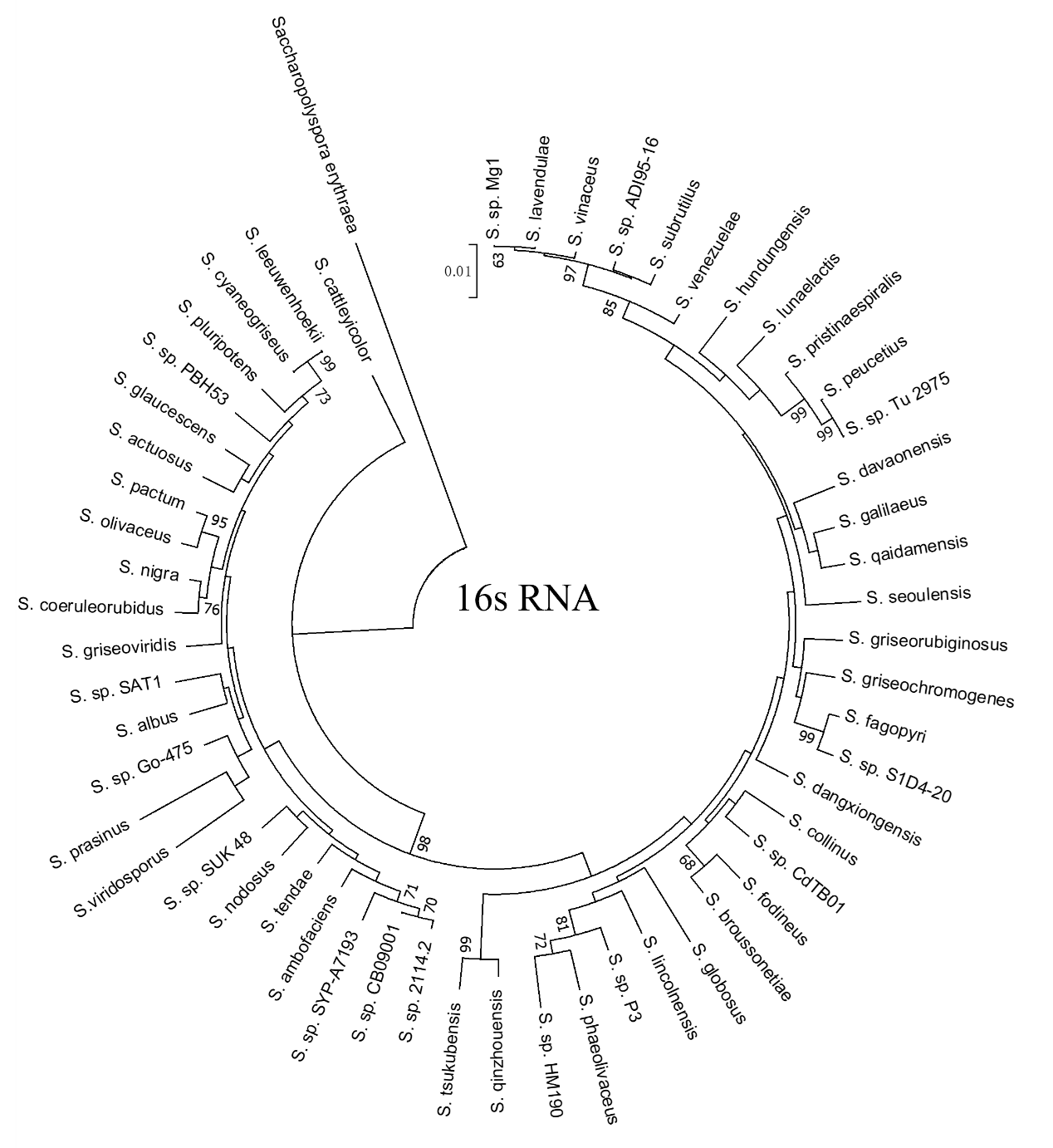


**Fig. S2** Phylogenetic analyses of strains with SLCG_7083-like proteins. Neighbor-joining phylogeny of 56 streptomyces based on their 16S sRNA sequences. The GenBank accession numbers of 16S sRNA genes and 56 LmbU-like proteins are listed in Supplementary **Table S1**.


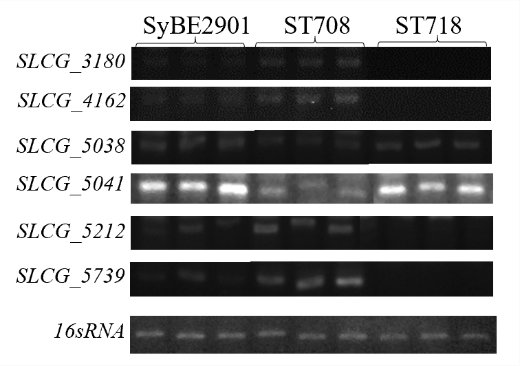


**Fig. S3** Semi-quantitative PCR of six differential expressed genes in strains SyBE2901, ST708 and ST718. In each case the experiments were repeated three times.

**Fig. S4** Amino acid sequence alignment of SLCG_3180 with Stage II sporulation protein E (SpoIIE, pfam07228).

**Table S1** The actinomycetes with 16S rRNA genes and SLCG_7083-like proteins used for constructing the phylogenetic trees in Fig. S1 and Fig. S2.

| Abbreviation | Accession number of 16S rRNA gene | SLCG_7083-like protein(s) | Species name |
| --- | --- | --- | --- |
| S. actuosus | NZ_CP029788.1 | WP_110633206.1 | *Streptomyces actuosus* ATCC 25421 |
| S. albus | NZ_CP010519.1 | WP_040246165.1 | *Streptomyces albus* DSM 41398 |
| S. ambofaciens | NZ_CP012382.1 | WP_053139827.1 | *Streptomyces ambofaciens* ATCC 23877 |
| S. cattleyicolor | NC_016111.1 | WP_014151358.1 | *Streptomyces cattleyicolor* NRRL 8057 =DSM 46488 plasmid pSCATT |
| S. coeruleorubidus | NZ_CP023694.1 | WP_150484179.1 | *Streptomyces coeruleorubidus* ATCC 13740 |
| S. collinus | NC_021985.1 | WP_020943313.1 | *Streptomyces collinus* Tu 365 |
| S. cyaneogriseus | NZ_CP010849.1 | WP_044385964.1 | *Streptomyces cyaneogriseus subsp. noncyanogenus* NMWT 1 |
| S. davaonensis | NC_020504.1 | WP_015656540.1 | *Streptomyces davaonensis* JCM 4913 |
| S. galilaeus | NZ_CP023703.1 | WP_150473632.1 | *Streptomyces galilaeus* ATCC 14969 |
| S. glaucescens | NZ_CP009438.1 | WP_043505237.1 | *Streptomyces glaucescens* GLA.O |
| S. globosus | NZ_CP030862.1 | WP_114055006.1 | *Streptomyces globosus* LZH-48 |
| S. griseochromogenes | NZ_CP016279.1 | WP_067303896.1 | *Streptomyces griseochromogenes* ATCC 14511 |
| S. griseorubiginosus | NZ_CP041168.1 | WP_141207866.1 | *Streptomyces griseorubiginosus* BTU6 |
| S. griseoviridis | NZ_CP034687.1 | WP_127176762.1 | *Streptomyces griseoviridis* F1-27 |
| S. hundungensis | CP032698 | AYG79379.1 | *Streptomyces hundungensis* BH38 |
| S. incarnatus | CP011497.1 | AKJ09459.1 | *Streptomyces incarnatus* NRRL 8089 |
| S. lavendulae | NZ_CP024985.1 | WP_037687881.1 | *Streptomyces lavendulae subsp. lavendulae* CCM 3239 |
| S. leeuwenhoekii | NZ_LN831790.1 | WP_029386517.1 | *Streptomyces leeuwenhoekii* |
| S. lincolnensis | NZ_CP022744.1 | WP_067442892.1 | *Streptomyces lincolnensis* |
| S. lunaelactis | NZ_CP026304.1 | WP_108155078.1 | *Streptomyces lunaelactis* MM109 |
| S. nigra | NZ_CP029043.1 | WP_108710919.1 | *Streptomyces nigra* 452 |
| S. nodosus | NZ_CP023747.1 | WP_107070579.1 | *Streptomyces nodosus* ATCC 14899 |
| S. olivaceus | NZ_CP016795.1 | WP_107060745.1 | *Streptomyces olivaceus* KLBMP 5084 |
| S. pactum | NZ_CP019724.1 | WP_079160052.1 | *Streptomyces pactum* ACT12 |
| S. peucetius | NZ_CP022438.1 | WP_100110185.1 | *Streptomyces peucetius subsp. caesius* ATCC 27952 |
| S. pluripotens | NZ_CP022433.1 | WP_043432231.1 | *Streptomyces pluripotens* MUSC 137 |
| S. prasinus | NZ_CP023697.1 | WP_095534489.1 | *Streptomyces prasinus* ATCC 13879 |
| S. pristinaespiralis | NZ_CP011340.1 | WP_078535467.1 | *Streptomyces pristinaespiralis* HCCB 10218 |
| S. puniciscabiei | NZ_CP017248.1 | WP_069783424.1 | *Streptomyces puniciscabiei* TW1S1 |
| S. qaidamensis | NZ_CP015098.1 | WP_062930261.1 | *Streptomyces qaidamensis* S10 |
| S. qinzhouensis | NZ_CP042266.1 | WP_146483215.1 | *Streptomyces qinzhouensis* SSL-25 |
| S. seoulensis | NZ_CP032229.1 | WP_031183566.1 | *Streptomyces seoulensis* KCTC 9819 |
| S. sp. 2114.2 | NZ_LT629768.1 | WP_162495284.1 | *Streptomyces* sp. 2114.2 |
| S. sp. ADI95-16 | NZ_CP033581.1 | WP_123080986.1 | *Streptomyces* sp. ADI95-16 |
| S. sp. CB09001 | NZ_CP026730.1 | WP_115745301.1 | *Streptomyces* sp. CB09001 |
| S. sp. CdTB01 | NZ_CP013743.1 | WP_058921990.1 | *Streptomyces* sp. CdTB01 |
| S. sp. Go-475 | NZ_CP026121.1 | WP_114256479.1 | *Streptomyces* sp. Go-475 |
| S. sp. GY16 | NZ_CP045096.1 | WP_152172733.1 | *Streptomyces* sp. GY16 |
| S. sp. HM190 | NZ_CP047318.1 | WP_159775126.1 | *Streptomyces* sp. HM190 |
| S. sp. Mg1 | CP011664.1 | AKL70349.1 | *Streptomyces* sp. Mg1 |
| S. sp. P3 | NZ_CP028369.1 | WP_107446893.1 | *Streptomyces* sp. P3 |
| S. sp. PBH53 | CP011799.1 | AKN70702.1 | *Streptomyces* sp. PBH53 |
| S. sp. QMT-28 | NZ_CP045643.1 | WP_153287694.1 | *Streptomyces* sp. QMT-28 |
| S. sp. S1D4-20 | NZ_CP041609.2 | WP_143614038.1 | *Streptomyces* sp. S1D4-20 plasmid pS1D4-20.1 |
| S. sp. SAT1 | NZ_CP015849.1 | WP_064536229.1 | *Streptomyces* sp. SAT1 |
| S. sp. SUK 48 | NZ_CP045740.1 | WP_153812498.1 | *Streptomyces* sp. SUK 48 |
| S. sp. SYP-A7193 | NZ_CP045547.1 | WP_153176893.1 | *Streptomyces* sp. SYP-A7193 |
| S. sp. T44 | NZ_CP047020.1 | WP_158927334.1 | *Streptomyces* sp. T44 |
| S. sp. Tu 2975 | NZ_CP047140.1 | WP_159684150.1 | *Streptomyces* sp. Tu 2975 |
| S. sp. Z022 | NZ_CP033073.1 | WP_121785966.1 | *Streptomyces* sp. Z022 |
| S. subrutilus | NZ_CP023701.1 | WP_150521766.1 | *Streptomyces subrutilus* ATCC 27467 |
| S. tendae | NZ_CP043959.1 | WP_150154757.1 | *Streptomyces tendae* 139 |
| S. tsukubensis | NZ_CP020700.1 | WP_130584731.1 | *Streptomyces tsukubensis* NRRL 18488 |
| S. venezuelae | NZ_CP029190.1 | WP_150260365.1 | *Streptomyces venezuelae* ATCC 21782 |
| S. vinaceus | NZ_CP023692.1 | WP_150492908.1 | *Streptomyces vinaceus* ATCC 27476 |
| S. viridosporus | NZ_CP023700.1 | WP_004980711.1 | *Streptomyces viridosporus* T7A ATCC 39115 |
| Saccharopolyspora erythraea | NZ_CP069353.1 | / | *Saccharopolyspora erythraea* NRRL 2338 |

**Table S2** Primers used in this study

| Primers | Sequence (5’-3’) |
| --- | --- |
| For construction of *SLCG_7083* inactivation | |
| 7083gRNA-F | GGACTAGT**GCCGATGCTGGCGCACTTCG**GTTTTAGAGCTAGAAAT |
| gTEMdn | CTCAAAAAAAGCACCGACTCGG |
| 7083up(gRNA)-F | *AAGTGGCACCGAGTCGGTGCTTTTTTTGAG*CGAGGACTTCACCGCCACG |
| 7083up-R | *ACCACGCTGAGGTGGCCCGCC*TGAAATGCCATCGGGAG |
| 7083down-F | *CTCCCGATGGCATTTCAGGC*GGGCCACCTCAGCGTGGT |
| 7083down-R | CCCAAGCTTCGACAGGAAGGGGAACAGCC |
| Q7083Y-F | GTTGCTGACCCTGGTGGACG |
| Q7083Y-R | TGACCGCAGGCGTGGTAGAG |
| For construction of *SLCG_7083* complementation | |
| 7083-F: | CCCAAGCTTCCGATGGCATTTCAGGCAGG |
| 7083R-R: | GGAAGATCTGCACTGTAGGGAAACGGGGC |
| For semi-quantitative RT-PCR | |
| SLCG_3180F | TATCCGCAGGCAGGCCATCT |
| SLCG_3180R | CCGCTCCTTCGGTCTTCTCG |
| SLCG4162-2F | CGAGTCCGTTCAGGCAGAGG |
| SLCG4162-2R | CGAGGTGGGCGTCGAAGTC |
| SLCG_5038F | GCGCTGCTCTTCGACATGGA |
| SLCG_5038R | GCTCGGCTTGCCCTCACG |
| SLCG_5041F | CCGTTGCCCCAGTCCCTC |
| SLCG_5041R | GCAGCGGACTCGTGGCGT |
| SLCG_5215F | GCGATGCCGCTGTTCCTGT |
| SLCG_5215F | GGCACTCCAGCCGACCTTG |
| SLCG_5739F | GGCGGAGGAGCGGTTCAA |
| SLCG_5739R | GCAGTGCCGTCGTGATGAGC |
| 16s-F | GCAATCTGCCCTTCACTCTGG |
| 16s-R | CTTCGTCCCAATCGCCAGTC |

The underlined sequence represents the restriction sites. Sequences in bold represent the guide sequence of sgRNA used in *SLCG_7083* gene deletion plasmid construction.
